# Collocations in Parsing and Translation

**DOI:** 10.3389/frai.2022.765695

**Published:** 2022-03-02

**Authors:** Eric Wehrli

**Affiliations:** Department of Linguistics, Université de Genéve, Geneva, Switzerland

**Keywords:** collocation, parsing, translation, deep syntax, multi-word expression

## Abstract

Proper identification of collocations (and more generally of multiword expressions (MWEs), is an important qualitative step for several NLP applications and particularly so for translation. Since many MWEs cannot be translated literally, failure to identify them yields at best inaccurate translation. This paper is mostly be concerned with collocations. We will show how they differ from other types of MWEs and how they can be successfully parsed and translated by means of a grammar-based parser and translator.

## 1. Introduction

Proper identification of collocations and more generally of multiword expressions (MWEs), is of critical importance for many NLP applications, notably translation. Most MWEs do not translate literally, therefore failure to detect them is likely to lead to inaccurate translation. This paper will mostly be concerned with collocations and will show how they differ from other types of MWEs and how they can be successfully parsed and translated by means of a grammar-based parser and translator.

Expressions which are frozen, such as so-called “words with spaces” (e.g., *by the way, close call, little by little*) function like lexical units and can simply be listed in the lexicon along with simple words. On the other hand, some other types of expressions show a sometimes very large degree of syntactic flexibility as illustrated with verbal collocations in Example (1) below, whose constituents are in boldface:

(1) a. The Bangkok stockmarket plunged 4.5% in a single day after **news** of the possible human-to-human transmission **broke**.
b. The top 500 listed firms **made** about 45% of the global **profits** of all American firms.
c. That **gave** the thoroughbred industry a needed **boost**.
d. Skeptics will wonder if the **money** will be efficiently and honestly **spent**.
e. This **record** will be hard to **break**.

Example (1a) shows a subject-verb collocation and (Examples 1b–c) verb-object collocations. In each of them, several words separate the two constituents of the collocation. Examples (1d–e) display the verb-object collocation *spend-money* and *break-record*, but because of syntactic transformations—passive in Example (1d), so-called *tough*-movement in Example (1e)—the two terms are in reverse order. Such examples clearly show the usefulness of syntactic knowledge for a precise identification of collocations.

The paper is organized as follows: in the next section, we will briefly review the main distinctions between the most common types of MWEs. Section 3 will focus on the parsing process and show how collocations can be useful with respect to categorial disambiguation. Section 4 is devoted to the translation process. We will first describe how the translation procedure handles collocations and then show how collocations are often useful for word sense disambiguation. Regarding this latter point, consider examples of adjective-noun collocations such as *standing* ***room***, *significant* ***lead***, *cold* ***case*** or *loose* ***change***. Though based on highly ambiguous nouns (in bold face), those collocations are rather unambiguous.

## 2. Collocations and multiword expressions

Multiword expressions can be defined as complex lexical units made of more than one word (see Wehrli, 2000, 2013; Sag et al., 2002; Seretan, 2011; Constant et al., 2017, among many others), where **word** is taken, very crudely, as a minimal string of letters between spaces (or some punctuation characters), and **lexical unit** corresponds to a syntactic or semantic unit. The following examples illustrate the diversity of (English) multiword expressions, and the discussion below will make those definitions clear.

(2) a. death penalty
b. by and large
c. to put off
d. carpe diem
e. to make an appointment
f. to pull one's leg

Example (2a) illustrates a noun-noun collocation, Example (2b) a fixed compound, Example (2c) a phrasal verb, Example (2d) a (Latin) proverb, Example (2e) a verb-object collocation, and Example (2f) an idiom.

In this paper, we adopt the classification proposed by Wehrli (2013)—influenced by earlier work (cf. Wehrli, 2000) as well as by Sag et al. (2002) and many others—with the following partition of multiword expressions:

- compounds (“word with spaces”)
*by and large, little by little, more or less, fr. fer à cheval, horse shoe*
- discontinuous words (e.g., phrasal verbs, i.e., verbs with particles in English or German, pronominal verbs in Romance)
  *she* ***looked*** *this word* ***up***
  *de. der Zug* ***fährt*** *um halb acht* ***ab*** (the train leaves at half past seven)
  *fr. l'homme* ***s'**est* ***suicidé*** “the man committed suicide”
- named entities
*John F. Kennedy, European Central Bank, World Economic Forum*
- idiomatic expressions
  *to kick the bucket, bouffer du lion* (to be hyperactive), *es. meter la pata* (to make a blunder)
- collocations
*hot topic, occupational hazard, fr. risques du métier, black economy, cold case*
  *to command admiration, to take up a challenge, to claim the life* *state of emergency, bone of contention, it. casco di banane* (*bunch of bananas*)
- other fixed expressions, proverbs, etc.
  *carpe diem, last but not least*, fr. à* plus ou moins brève échéance, sooner or later, a pain in the neck*.

From a syntactic viewpoint, compounds and named entities are lexical units of lexical category (noun, adjective, adverb, etc.). They behave just like simple lexical items (words) but happen to contain spaces (or sometimes punctuation signs). We will consider that they belong to the lexical database^1^. Discontinuous words (e.g., phrasal verbs) can also be considered as lexical units of lexical category (verbs in our examples), which happen to be made of two parts—the verb and the particle—which may not be adjacent to each other. It is the parser's task to recognize that the two elements belong to the same lexical unit^2^.

In contrast to compounds, named entities and discontinuous words, collocations and idiomatic expressions at the syntactic level do not behave like lexical units but rather like syntactic units (phrases). They constitute noun phrases in the case of noun-noun, adjective-noun or noun-preposition-noun collocations, verb phrases in the case of verbal collocations (verb-direct object, verb-prepositional object, etc.). Such MWEs must also be listed in the lexical database used by the parser—they cannot be guessed—for instance as associations of two lexemes (or *groupements usuels* “usual phrases” as coined by Bally, 1909).

While many of our remarks and observations hold for all or many of the MWEs subclasses, we will mostly be concerned with collocations, taken here broadly as the association of two lexical units in a particular grammatical configuration. While idiomatic expressions often display semantic opacity (e.g., *to kick the bucket* in the sense of *dying*) as well as restrictions on their syntactic behavior, such as no passive, no movement, no modifier, etc., the constituents of a collocation usually keep their usual syntactic properties, and are semantically relatively transparent.

### 2.1. Multiword Expressions Matter for NLP

The importance of MWEs for NLP applications, such as translation, is widely recognized^3^. To understand why, consider the three following points:

- most expressions cannot be translated literally
  (*dead loss, to make an appointment, to kick the bucket*)
- some compounds as well as some fixed expressions do not respect grammatical rules, e.g., *by and large*
- MWEs have a high frequency named entities constitute approximately 10% of newspaper articles, and very few sentences do not contain any compound or collocation.

As already pointed out, it is therefore necessary for most NLP applications to “know” and to properly identify MWEs. This, however, may turn out to be a complicated task if you consider what I will refer to as the syntactic flexibility of many MWEs, limited here (for collocations) to the three following cases (see Sag et al., 2002):

- Adjectival or adverbial modifiers can often occur within a collocation, in-between the two terms, e.g., *a* ***school*** *of little* ***fishes***
- Several types of collocations can undergo grammatical processes which may modify the canonical order of the collocation (e.g., passive, relativization, etc.)
- Occasionally, a noun in a verb-object or subject-verb collocation can be replaced by a pronoun.

Syntactic flexibility is particularly important with verbal collocations such as verb-object or verb-prepositional object and subject-verb, where the two terms of the collocation can be separated by an arbitrary number of words; due to syntactic transformations, such as passive, relativization, interrogation, etc., they can also occur in a reverse order, which of course makes it difficult to identify them in a sentence. To illustrate, consider the following examples, in which constituents of collocations are in boldface.

(3) a. The scheme **addresses** one of America's prickliest **problems**.
b. The **problem**—that poor children do not get the chances that rich ones do—is a real one, but needs to be **addressed** earlier.
c. The Bangkok stockmarket plunged 4.5% in a single day after **news** of the possible human-to-human transmission **broke**.

Sentence (Example 3a) contains a verb-direct object collocation (*to address a problem*) with several words in-between the two terms *addresses* and *problems*. The same collocation occurs in sentence (Example 3b), where the two terms are in reverse order due to passive and are separated by considerable material. Finally, sentence (Example 3c) illustrates a subject-verb collocation (*the news breaks*)—a collocation type much less frequent than the verb-object type—with again several words separating the two terms.

Another transformation can affect collocations, pronominalization, as in the Example (4) below. Each of the two sentences in Example (4a) contains an occurrence of the collocation *to make a case*. Notice, however, that in the second sentence, the direct object (*case*) has been pronominalized. In other words, the pronoun *it*, which refers to the noun *case* of the previous sentence, validates the collocation. The second (Example 4b) illustrates a similar scenario, with the pronoun *it* referring to the noun *money*. The pronoun occurs in the subject position of the passive clause *would be well spent*, it is therefore interpreted as direct object of the verb, corresponding to an occurrence of the collocation *to spend money*.

(4) a. Every Democrat is **making** this **case**. But Mr Edwards **makes it** much more stylishly than Mr Kerry.
b. …though where the **money** would come from, and how to ensure that **it** would be well **spent**, is unclear.

## 3. Treatment of MWEs in a linguistically-based system

This section briefly describes the way collocations and other types of MWEs are handled by Fips, our multilingual parser^4^. As already mentioned, we consider that MWEs must be “known” by the system, that is they are listed in the lexical database available to the parser, either as words with spaces (e.g., compounds) or as an expression, for instance in the case of collocations, as an association of two lexical items. Compounds (and listed named entities) can be recognized just like plain words, as soon as the parser reads them. As for collocations, because their detection requires syntactic knowledge, they are handled during the syntactic analysis, as soon as the last term of the expression (collocation or expression) is added to the structure^5^.

To achieve this goal, we added a collocation database to each of the monolingual lexical databases, using the system for collocation extraction developed by Violeta Seretan and others at LATL (cf. Seretan and Wehrli, 2009; Seretan, 2011). This system extracts candidate-collocations from a corpus, filters those candidates using standard association measures. A linguist/lexicographer can then select the best candidates to be added to the collocation database. Entries in the database specify the two lexical items which form the collocation, as well as some additional information such as the type of collocation (e.g., noun-noun, verb-object, etc.) and the possible presence of a preposition (e.g., noun-preposition-noun, verb-preposition-object). The current content of the databases for five European languages is shown in Table 1 below.

**Table 1:** Number and types of collocations in the Fips lexical database.

**Collocation type**	**English**	**French**	**German**	**Italian**	**Spanish**
Adjective-noun	3,510	9,918	617	1,518	1,689
Noun-noun	6,207	527	3,253	162	77
Noun-prep-noun	652	10,753	30	1,501	1,106
Verb-object	1,050	2,287	247	339	1,102
Others	1,177	3,911	461	450	597
Total	12,596	26,873	4,608	3,808	4,571

The procedure responsible for the identification of collocations in the Fips parser works as follows. During the parse, it is triggered by the application of a right (or left) attachment rule. Governing nodes (i.e., dominating nodes) of the attached element are iteratively considered, stopping when a node of major category is reached (NP, AP, VP, AdvP)^6^. Then, the procedure checks whether the pair [governing item + governed item] corresponds to an entry in the collocation database.

As an illustration, consider the following simple example. We will show more complex cases below, which will require a refinement of the procedure.

**Figure 1:**
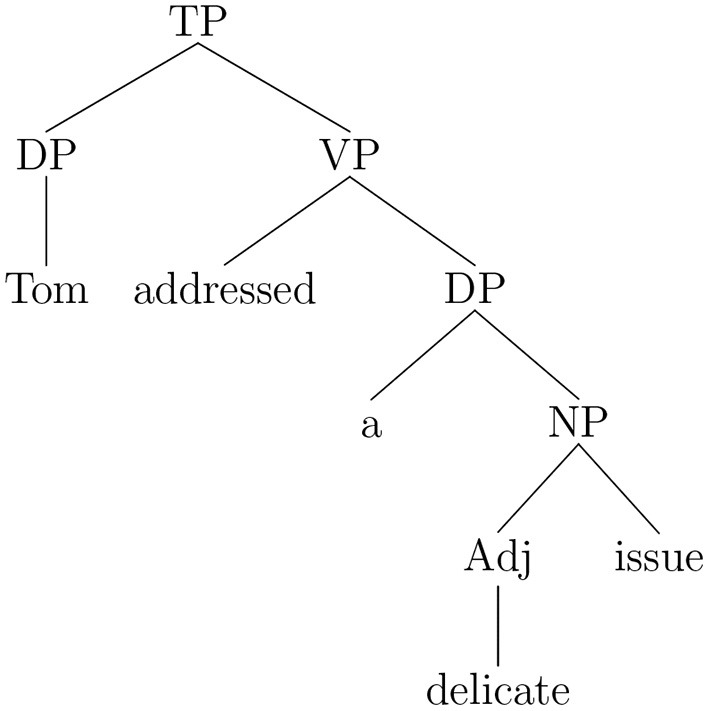
Phrase-structure representation for sentence (Example 5a).

Consider first the sentence (Example 5a) with the verb-object collocation *to address an issue*. The structure assigned by the Fips parser, is given in Example (5b) in the labeled-bracketing notation. Figure 1 represents the structure in the more familiar phrase-structure representation.

(5) a. Tom addressed a delicate issue
b. [_TP_ [_DP_ Tom ] [_VP_ addressed [_DP_ a [_NP_ [_Adj_ delicate ] issue ] ] ] ]

When the parser comes to the word *issue*, a left-attachment rule creates the noun phrase [_NP_ [_Adj_ delicate ] issue ], which in turn is attached by a right-attachment rule to the determiner phrase headed by the indefinite determiner *a* already attached as direct object of the verb *addressed*. Following the collocation identification procedure described above, we consider iteratively the nodes dominating the noun phrase *issue*. The first dominating node is the DP node, which is not a major category node, and then the VP node, which is a major category node. Therefore, the procedure halts and verifies if the pair [*addressed* + *issue*], with *address* as a verb and *issue* as a direct object noun, corresponds to an entry in the collocation database. This being the case, the collocation reading is assigned to the verb phrase.

Consider now some more complex cases, when a collocation element undergoes a syntactic movement, as in Examples (6a–e).

(6) a. *wh*-interrogatives
Which **record** did Tom **break**?
[_CP_ [_DP_ which record]*i* did [_TP_ Tom [_VP_ break [_DP_ e]*i* ] ] ]
b. relative clauses
The **record** that Tom tried to **break** was very old.
c. *tough*-movement
These **records** seem relatively easy to **break**.
d. *wh*-interrogative + *tough*-movement
Which **record** did Tom consider most difficult to **break**?
e. left dislocation + small clause + *tough*-movement
fr. Ce **record**, Tom le considère très difficile à **battre**
“this record Tom considers very difficult to break”
[_CP_ [_DP_ ce record]*i* [_TP_ Tom le*i* considère [_DP_ e]*i*
[_FP_ [_DP_ e]*i* difficile [_CP_ à [_TP_ [_VP_ battre [_DP_ e]*i* ] ] ] ] ] ]

How can the parser detect the collocation *to break a record* in those examples? The answer is indeed surprisingly simple if you consider that Fips assumes Chomsky's *wh*-movement analysis for such sentences (cf. Chomsky, 1977, 1981). According to this analysis, *wh*-phrases such as interrogative phrases or relative pronouns bind an empty category in their canonical position, that is the position corresponding to their interpretation^7^. For instance, a *wh*-phrase interpreted as a direct object binds an empty category in the post-verbal position. In the structure (Example 6a), the empty category associated with the *wh*-phrase. The link between the two constituents is expressed by the shared index *i*.

In order to correctly identify expressions with *wh*-complements (in our examples, *wh*-objects), the above procedure must be slightly altered so that it is triggered not only by the attachment of lexically realized complements (as in Example 5), but also by the attachment of an empty category (a trace) in complement position of a verb (or of an adjective). In the latter case, the procedure verifies that the verb (or the adjective) and the antecedent of the complement correspond to an entry in the collocation database.

Let us now turn to the much more intricate case of collocations whose direct object (or subject) has been pronominalized, as in Examples (7a–b) and (8a–b).

(7) a. Tom set a new **record** last year and he hopes to **break it** this year.
b. Tom set a new **record** last year. He hopes to be able to **break it** again.

(8) a. The **news** had been expected for a long time and **it** finally **broke** last night.
b. The **news** had been expected for a long time. **It** finally **broke** last night.

In such cases, the detection of the collocation depends on the identification of the antecedent (the referent) of the pronoun. Fips uses an anaphora resolution component which tries to associate a pronoun with a preceding noun phrase in the same sentence as in Examples (7a) and (8a), or in the preceding sentence as in Examples (7b) and (8b)^8^. The collocation identification procedure has been updated, again, in order to be triggered by a pronoun attached, for instance, in the direct object position as in Example (7) or in subject position as in Example (8). First the anaphora procedure attempts to identify the antecedent of the pronoun and then the collocation procedure verifies whether the verb and the antecedent of the pronoun correspond to a collocation listed in the database.

## 4. Translating collocations

ITS-2 (Wehrli et al., 2009) is a rule-based automatic translator based on the Fips parser. The translation process adopts the classical three-phase scheme of a transfer system: parsing, transfer and generation. First, the input sentence is parsed, producing an information-rich representation of the sentence structure with predicate-argument labels. Then, the transfer module maps this abstract source language representation to a target language representation, traversing the source structure in the following order: head, left subconstituents, right sub-constituents. The lexical transfer takes place during the transfer of the head and produces an equivalent term in the target language, of identical or different category. The structure of the target language is projected on the basis of each lexical head. In this way, the final result reflects the lexical characteristics of the target language. The categorical nature of the arguments, on the other hand, is determined by the lexical properties of the target language predicate (i.e., its subcategorization features). The necessary information is available in the lexical database. The generation module also includes syntactic procedures (transformations) that move constituents (e.g., *wh*-movement) or modify the structure of arguments (passive, causative, etc.), or in the case of Romance languages, cliticize pronominal arguments. Finally, the morphology procedure of the target language is applied to give words their final form.

Currently, the system translates between five languages distributed into the following four main pairs: {English, Italian, German, Spanish} ↔ French. For each language pair, a bilingual dictionary implemented as a relational table specify the associations between source language and target language lexical items. Entries in the bilingual dictionary also contain information such as translation context, semantic descriptors and argument matching for predicates such as verbs and adjectives.

As mentioned in the previous section, Fips roughly follows the linguistic assumptions of Chomsky's generative grammar, along with concepts from other generative theories such as LFG (Bresnan, 2001) and Simpler Syntax (Culicover and Jackendoff, 2005).

All the structures built by both the parser and the translator follow the model given in the scheme (Example 9), where **X** is a (possibly empty) lexical head, **XP** is the phrasal category projected on the basis of the head **X**, while **L** and **R** represent, respectively, (zero or more) left and right subconstituents.

(9) [_XP_ L X R ]

In this schema, **X** is a variable which takes its value in the set of lexical categories (**N**oun, **A**djective, **V**erb, **D**eterminer, **P**reposition, **Adv**erb, **C**onjunction, **Inter**jection) to which we add two additional categories, **T**ense and **F**unction. **TP** is the phrasal node that dominates the tense marker (T); it roughly corresponds to the traditional **S** sentential node used in many grammatical formalisms. **FP** constituents are secondary predicative structures of nominal, adjectival or prepositional flavor. They correspond to the “small clause” structures of standard generative grammar (cf. Haegeman, 1994). As already mentioned, this is a rather minimal variant of the X̄ theory with just two levels, the head and the maximal projection.

Notice that contrary to common views in recent generative developments (e.g., Chomsky's Minimalism), the constituent structures returned by the parser or generated by the translator are not strictly binary. Also, rather than **Spec**ifier and **Compl**ement, we use the more neutral **L**eft and **R**ight to refer to subconstituents. The motivation behind this choice is the observation that across languages, complements can sometimes occur as left subconstituents, right subconstituents or even distributed in both positions. Similarly non complements, such as adverbial and adjuncts can also occur either to the left of the head, to its right or both.

### 4.1. Collocation Identification

Arguably, the most challenging task in the treatment of collocations in the translation process is their proper identification. As we have seen, for several types of collocations, in particular for verbal collocations, the two lexical units of the collocation can be several words apart and may not even be in the expected order, due to grammatical processes such as *wh*-fronting, passivization and others. In extreme cases, the distance between the two lexical units can exceed dozens of words. It may also be the case that they do not strictly speaking cooccur within the same sentence, as in Example (10b), with the two lexemes in bold face:

(10) a. In 1935, Jesse Owens set a long jump world **record** that was not **broken** until 1960 by Ralph Boston.
b. The **record** was set in 2003. It is likely to be **broken** during the next Olympics.

Notice that in order to adequately handle such sentences, a comprehensive syntactic analysis is necessary, capable of interpreting fronted elements, such as the relative pronoun in Example (10a), intra-sentential pronominal reference, as in the case of a relative pronoun and its antecedent in the same sentence, as well as (at least some) extra-sentential pronominal reference, as in Example (10b), where the pronoun *it* which acts as (surface) subject of the second sentence refers to the noun *record* occurring in the preceding sentence. Given the fact that *it* is the subject of a passive sentence with verb *break*, we have an occurrence of the *break-record* verb-object collocation.

To put it in a slightly different way, in order to correctly perform the identification of the collocation *break-record* in sentence (Example 11a), the parser must be able to (i) recognize the presence of a relative sentence, (ii) determine the role of the relative pronoun with respect to the verb of the relative sentence (direct object), and (iii) identify the referent (antecedent) of the relative pronoun.

(11) a. The record that Tom had broken.
b. [_DP_ the [_NP_ record*i* [_CP_ that*i* [_TP_ [_DP_ Tom ] had [_VP_ broken [_DP_ e]*i* ] ] ] ] ]

These three tasks are accomplished by the Fips parser, which assigns to the sentence (Example 11a) the syntactic structure (Example 11b), in which the index i indicates that the three constituents form a chain that links the noun *record* to the direct object position of the verb form *broken*.

An important function of a “deep” syntactic parser is to establish a syntactic normalization of the sentence, i.e., a canonical way of representing the basic structure of a sentence, abstracting away from surface differences due to grammatical (or stylistic) processes. Examples of standardized structures commonly used in generative grammar are the so-called traces, that is co-indexed empty categories in argument positions, or functional structures. For the task of collocation identification, normalization is particularly useful in the sense that it provides an abstract, unified and standardized representation on which the presence (or absence) of a collocation can be easily determined. To illustrate this point, consider the following example, which contains two collocations *to set a deadline* and *to meet a deadline*, both of them recognized by the parser:

(12) a. The *deadline* that we had *set* could not be *met*.
b. [_TP_ [_DP_ the [_NP_ deadline*i, j* [_CP_ [_DP_ e]*i* that [_TP_ [_DP_ we ] had [_VP_ set [_DP_ e]*i* ] ] ] ] could [_VP_ not be [_VP_ met [_DP_ e]*j* ] ] ] ]

As shown by the structure (Example 12b), the subject of the main sentence *deadline* is the head of a double chain represented by the indices **i** and **j**, respectively. The first chain describes the relation between the head of the relative sentence and the direct object position of the embedded verb *set* (via the null relative pronoun), while the second chain represents the promotion of the direct object of the main verb *met* to the subject position, characteristic of the passivation process. Thanks to the normalization performed by the parser, which in a way “undoes” the above mentioned grammatical processes, the task of detecting the presence of a verb-object collocation is made considerably easier.

### 4.2. Collocation Transfer and Generation

When a collocation is identified in a source language sentence, all its elements are assigned a “collocation constituent” feature which will block their automatic literal translation. Rather, the lexical transfer procedure will look for an entry in the bilingual database for that collocation. If none is found, the literal translation will apply. On the other hand, if an entry is found, two different situations can arise: (i) the corresponding target language lexical unit is a simple lexeme. In this case, the syntactic head of the collocation (in our verb-object example, the verb) is translated by means of that lexeme. In the more interesting case (ii), the corresponding target language lexical item is itself a collocation. This is what happens with the *meet-deadline* example in an English to French translation. The bilingual dictionary specifies a correspondence between the English collocation *meet-deadline* and the French collocation *respecter-échéance*. Based on that information, *meet* will be translated as *respecter*, and the transfer module registers the fact that the lexical head of the argument corresponding to the direct object of the source language verb (in this case also a direct object) is the French lexeme é*chéance* (and not one the numerous possible other translations of the noun *deadline*.

As we have already seen, transfer produces an abstract representation of the target language, to which grammatical processes (passive, movement transformation, etc.) and morphological generation apply to create the target sentence. Unless particular restrictions are specified in their lexical entry, collocations are subject to the same grammatical and morphological processes as other lexical items.

Consider the various scenarios, such as source collocation to target lexeme (Example 13), source lexeme to target collocation (Example 14) and source collocation to target collocation (Example 15).

(13) fr. avoir envie (“have desire”) → to want
fr. avoir besoin (“have need”) → to need
fr. prendre garde (“take guard”) → to watch out
fr. prendre au piège (“take in trap”) → to trap

(14) to shadow/to tail → fr. prendre en filature (“take in spinning”)
awareness / realization → fr. prise de conscience
scenario → fr. cas de figure
perjury → fr. faux témoignage
to brief → fr. donner des instructions
de. frühstücken → to have breakfast

(15) to take a look → fr. jeter un coup d'oeil
to put on a show → fr. donner un spectacle
the tax base → fr. l'assiette fiscale
to bridge the gap → fr. combler le fossé
fr. la nouvelle est tombée → the news broke

As noted earlier, the general transfer algorithm recursively traverses the phrase structure generated by the parser in the following order: head, left subconstituents, right subconstituents. The lexical mapping between the source and target elements occurs when a non-empty head is transferred. At this point, the bilingual dictionary is accessed to retrieve the target language item that is associated with the source language lexical item. For instance, in the case of Example (16), the sequence of lexical transfer is given in Example (17):

(16) fr. Jean a mangé un biscuit (‘Jean has eaten a cookie')
(17) a → ∅, mangé → eat, Jean → Jean, un → a, biscuit → cookie

Returning to the three scenarios above, when a collocation is identified in the source sentence, lexical transfer occurs on the basis of the collocation. If, as in Example (13), the source collocation corresponds to a target language simple lexeme, that lexeme takes the place of the head of the target language phrase (in our examples, the verbal phrase) while the second term of the source collocation is ignored. In the other two scenarios, Example (14) where a source language lexeme is translated by means of a collocation and Example (15) where a source collocation is associated with a target collocation, the generation procedure creates the target phrase on the basis of the head of the collocation with the second term generated in its canonical position (e.g., as direct object in the case of a verb-object collocation, as a prepositional complement in the case of a noun-preposition-noun collocation, etc.).

Finally, consider Example (18) with a subject-verb collocation both in French (*la nouvelle tombe* “the news falls”) and in English *the news breaks*. Notice first that the French sentence contains the quasi-auxiliary verb *venir* (“to come”) used here to express the recent past and will be translated by the auxiliary *have just*.

(18) La nouvelle vient de tomber.
  the news comes to fall
  “the news has just broken”

Notice that in such examples collocation knowledge not only provides the proper translation of the verb (*tomber* is to be translated as *break* rather than *fall*), it also provides the proper translation for the noun (*nouvelle* is to be translated as *news* rather than *short story*). A large number of collocations involve highly polysemous words, but in the context of the collocation those words usually have an unambiguous meaning. In this respect, collocation knowledge can be viewed as an effective help to disambiguate otherwise polysemous words. This is true not only for nouns, as in Example (19) but also for other lexical categories, in particular adjectives:

(19) a significant **lead**
a high **resolution**
some loose **change**

In the three examples, the nouns in boldface, taken individually, are highly polysemous (for the first one even the pronunciation is unclear [li:d] vs. [led], about 10 different senses are listed in the Robert-Collins dictionary).

## 5. Conclusion

In this paper, we described a comprehensive multilingual translation system which combines a deep syntactic parser—including a collocation detection component and an anaphora resolution mechanism—using an information-rich lexical database including monolingual lexical units (lexemes and MWEs), as well as bilingual data, i.e., correspondences between lexical items of source and target languages. Multiword expressions and in particular collocations constitute an important aspect of natural language and must be treated adequately by natural language processing systems, not only because of the high frequency of MWEs in most documents but also, in the case of translation, because they usually cannot be translated literally. Hence, MWEs must be known by the translation system, i.e., listed in the lexical database, and reliably detected in the course of the source language analysis. As we have shown, this is by no means a trivial task, given the fact that the constituents of an expression can be arbitrarily far away from each other and, due to grammatical processes such as passivization or fronting, not even in the expected order. In all those cases, we argued that use of “deep” syntactic knowledge seems to be the surest way to identify MWEs. Such abstract syntactic representation is also useful for MWEs generation in the target language.

## Data Availability Statement

The original contributions presented in the study are included in the article/supplementary material, further inquiries can be directed to the corresponding author.

## Author Contributions

EW is fully responsible for the entire research and manuscript.

## Conflict of Interest

The author declares that the research was conducted in the absence of any commercial or financial relationships that could be construed as a potential conflict of interest.

## Publisher's Note

All claims expressed in this article are solely those of the authors and do not necessarily represent those of their affiliated organizations, or those of the publisher, the editors and the reviewers. Any product that may be evaluated in this article, or claim that may be made by its manufacturer, is not guaranteed or endorsed by the publisher.

## Footnotes

^1^In the case of named entities—a boundless class—most of them should probably be listed in domain-specific (or application-specific) lexicons. We will not pursue this matter further in this paper.

^2^Notice that the identification of pronominal verbs in German or in Romance languages is quite similar. Here too, we have a particular lexeme constituted of two elements—the verb and the pronoun—which may not be adjacent.

^3^There is an impressive amount of literature dedicated to MWEs. See in particular (Pastor et al., 2016; Sailer and Markantonatou, 2018; Parmentier and Waszczuk, 2019) or the numerous contributions associated with the European COST action PARSEME https://typo.uni-konstanz.de/parseme/index.php/2-general/49-publications. For phrasal verbs detection by means of a finite state parser, see Machonis (2018) and references given there. Collocations have also been extensively studied within the framework of the Meaning-Text theory (cf. Mel'čuk, 1996, 2003). MWEs are also extensively studied within the context of neural machine translation, see for instance (Park and Tsvetkov, 2019) and references given there.

^4^A detailed description of the Fips parser is given in Wehrli (2007) and Wehrli and Nerima (2015), which also describes the structure of the lexical database. See Wehrli et al. (2010); Wehrli (2014); Foufi et al. (2019), and Wehrli et al. (2020) for issues related to the treatment of collocation in parsing.

^5^Alternatively, one might consider that the identification could be delayed until the end of the parsing process. This, however, would prevent the parser from exploiting collocational knowledge, for instance as heuristics to rank alternatives.

^6^NP stands for “noun phrase,” AP for “adjectival phrase,” VP for “verb phrase,” and AdvP for “adverb phrase.” The Fips grammar also uses the labels DP for “determiner phrase,” PP for “prepositional phrase” and TP for “tense phrase”.

^7^In Chomsky's view, the *wh*-phrase moves from its “original” (also called canonical) position to the initial position of a sentence, leaving a trace (an empty category co-indexed with the trace) behind.

^8^See Mitkov (2002) for a thorough discussion of anaphora resolution. See Wehrli and Nerima (2013); Loáiciga and Wehrli (2015) for more details about the anaphora procedure developed for Fips, which owes much to the work of Hobbs (1978) and Lappin and Leass (1994).
